# The extra X chromosome and autoimmune susceptibility in Klinefelter syndrome

**DOI:** 10.1210/clinem/dgag268

**Published:** 2026-07-04

**Authors:** Elisa Gatta, Andrea Delbarba, Virginia Maltese, Caterina Buoso, Carlo Cappelli

**Affiliations:** Department of Clinical and Experimental Sciences, SSD Endocrinologia, University of Brescia, ASST Spedali Civili, Brescia 25121, Italy; Centro per la Diagnosi e Cura delle Neoplasie Endocrine e delle Malattie della Tiroide, University of Brescia, Brescia 25121, Italy; Department of Clinical and Experimental Sciences, SSD Endocrinologia, University of Brescia, ASST Spedali Civili, Brescia 25121, Italy; Department of Clinical and Experimental Sciences, SSD Endocrinologia, University of Brescia, ASST Spedali Civili, Brescia 25121, Italy; Department of Clinical and Experimental Sciences, SSD Endocrinologia, University of Brescia, ASST Spedali Civili, Brescia 25121, Italy; Department of Clinical and Experimental Sciences, SSD Endocrinologia, University of Brescia, ASST Spedali Civili, Brescia 25121, Italy; Centro per la Diagnosi e Cura delle Neoplasie Endocrine e delle Malattie della Tiroide, University of Brescia, Brescia 25121, Italy

**Keywords:** Klinefelter syndrome, autoimmunity, sex chromosome dosage, autoimmune disease, immune dysregulation, XXY syndrome

## Abstract

**Objective:**

To systematically evaluate and quantitatively synthesize the association between Klinefelter syndrome (KS) (47,XXY karyotype) and autoimmune susceptibility.

**Methods:**

PubMed/MEDLINE, Scopus, and Web of Science were systematically searched from database inception to April 30, 2026, for studies evaluating autoimmune diseases or autoimmune serological markers in individuals with KS. The protocol was registered in PROSPERO (CRD420261399097). Random-effects models were prespecified, summary data were extracted from published reports, and pooled effect estimates were expressed as odds ratios (ORs) with 95% CIs. Subgroup analyses were performed according to outcome definition (isolated autoantibody positivity vs clinically manifest autoimmune disease). Risk of bias was assessed using the Newcastle–Ottawa Scale.

**Results:**

Eight studies were included in the qualitative synthesis and six studies (8 datasets) in the quantitative analysis, comprising 2289 individuals with KS and 51 250 114 controls. KS was associated with markedly increased autoimmune susceptibility compared with control populations (OR 23.25, 95% CI 9.96-54.31), with moderate-to-substantial between-study heterogeneity (I^2^ = 66.4%). Associations were stronger for clinically manifest autoimmune diseases (OR 48.46, 95% CI 17.68-132.87) than for isolated autoantibody positivity (OR 9.99, 95% CI 1.18-84.77). The autoimmune spectrum predominantly involved female-predominant and interferon-associated disorders, including systemic lupus erythematosus, Sjögren syndrome, systemic sclerosis, autoimmune thyroid disease, and type 1 diabetes mellitus.

**Conclusion:**

KS is associated with increased autoimmune susceptibility, particularly for clinically overt autoimmune diseases. These findings support a potential contribution of sex chromosome complement to immune dysregulation and reinforce the importance of clinical awareness of autoimmune comorbidities in individuals with KS.

Klinefelter syndrome (KS) is the most common sex chromosome aneuploidy, affecting approximately 1 to 2 per 1000 male newborns, and is characterized by the presence of one or more supernumerary X chromosomes (1). The classic 47,XXY karyotype accounts for approximately 80% to 90% of KS cases, whereas the remaining 10% to 20% include higher-grade aneuploidies (eg, 48,XXXY, 49,XXXXY, and 48,XXYY), mosaic forms—most commonly 47,XXY/46,XY—and structurally abnormal X chromosomes such as 47,iXq,Y (2, 3).

KS is currently recognized as a multisystem disorder extending far beyond infertility and hypogonadism. In addition to hypergonadotropic hypogonadism and impaired reproductive function, patients frequently develop metabolic, cardiovascular, skeletal, neurocognitive, and immune-related complications (4, 5). Metabolic syndrome, insulin resistance, type 2 diabetes mellitus, visceral obesity, and dyslipidaemia are significantly more prevalent in KS compared with the general male population (6). Reduced bone mineral density and osteoporosis are also common, likely reflecting the combined effects of androgen deficiency and altered body composition (7-9). Moreover, increasing evidence suggests that the supernumerary X chromosome may contribute directly to immune dysregulation and chronic inflammation (10). Overall, these findings support the concept of KS as a complex systemic condition requiring multidisciplinary management throughout life (5).

Autoimmune diseases are conditions in which tissue damage results from T-cell or antibody-mediated immune responses directed against self-antigens. Traditionally, autoimmune diseases are defined according to the Witebsky–Rose criteria, which include the presence of an immune response against self-antigens, experimental reproduction of the disease in animal models, and transferability of the disease through serum or immunocompetent cells from affected individuals (11). Reflecting the growing recognition of autoimmunity as a major clinical entity, more than 100 distinct autoimmune disorders have been described to date (12).

The presence of the X and Y chromosomes determines biological sex differentiation. However, sex chromosomes also contribute to sexual dimorphism beyond gonadal development through dosage-dependent and epigenetic mechanisms affecting multiple physiological systems, including immunity (13). The marked female predominance observed in many autoimmune diseases has focused attention on the X chromosome as a potential regulator of immune tolerance and autoimmunity (14). Indeed, the X chromosome harbors several immune-related genes involved in lymphocyte development, cytokine signaling, and immune regulation, including *FOXP3* and genes associated with interleukin pathways (15, 16). Altered X chromosome dosage and epigenetic regulation may therefore promote immune dysregulation and increase susceptibility to autoimmune diseases, such as in Turner syndrome (17, 18).

Given the emerging role of the X chromosome in immune regulation and the unique genetic background of Klinefelter syndrome, characterized by the presence of a supernumerary X chromosome, KS represents a potentially informative human model for investigating the relationship between sex chromosome dosage and autoimmunity. Although accumulating evidence suggests increased autoimmune burden in KS, available studies remain fragmented across endocrine and systemic autoimmune phenotypes, preventing an integrated understanding of whether supernumerary X chromosome dosage contributes to a coherent autoimmune profile. Therefore, the present systematic review aimed to quantify autoimmune susceptibility in the classic 47,XXY karyotype and explore the potential contribution of supernumerary X chromosome dosage to immune dysregulation.

## Materials and methods

### Search strategy and inclusion criteria

We performed a systematic review of observational studies evaluating autoimmune diseases and autoimmune serological markers in Klinefelter syndrome. PubMed/MEDLINE, Scopus, and Web of Science were systematically searched from database inception to April 30, 2026.

The review question was structured according to the Population–Exposure–Outcome (PEO) framework to evaluate whether supernumerary X chromosome dosage in KS is associated with increased autoimmune burden.

The search strategy combined terms related to Klinefelter syndrome and autoimmune diseases, including: ((“Klinefelter Syndrome”[Mesh] OR Klinefelter* OR 47,XXY OR XXY) AND (“Autoimmune Diseases”[Mesh] OR autoimmun* OR autoimmune disease* OR autoantibod*)).

The search was restricted to studies published until April 30, 2026. Only articles published in English were considered. Preclinical studies, conference proceedings, reviews, editorials, and case reports were excluded. To minimize the risk of missing relevant studies, the reference lists of all included articles were also screened.

### Study selection

C.C and E.G. independently read the titles and abstracts of the records generated by the search algorithm. They then determined which studies were eligible based on predefined criteria. Disagreements regarding study eligibility were resolved through consensus discussion.

### Reporting

This systematic review followed the PRISMA (Preferred Reporting Items for Systematic Reviews and Meta-Analyses) guidelines (19). The corresponding PRISMA flow diagram is reported in Fig. 1. Risk of bias in observational studies was assessed using the Newcastle–Ottawa Scale (NOS).

**Figure 1:**
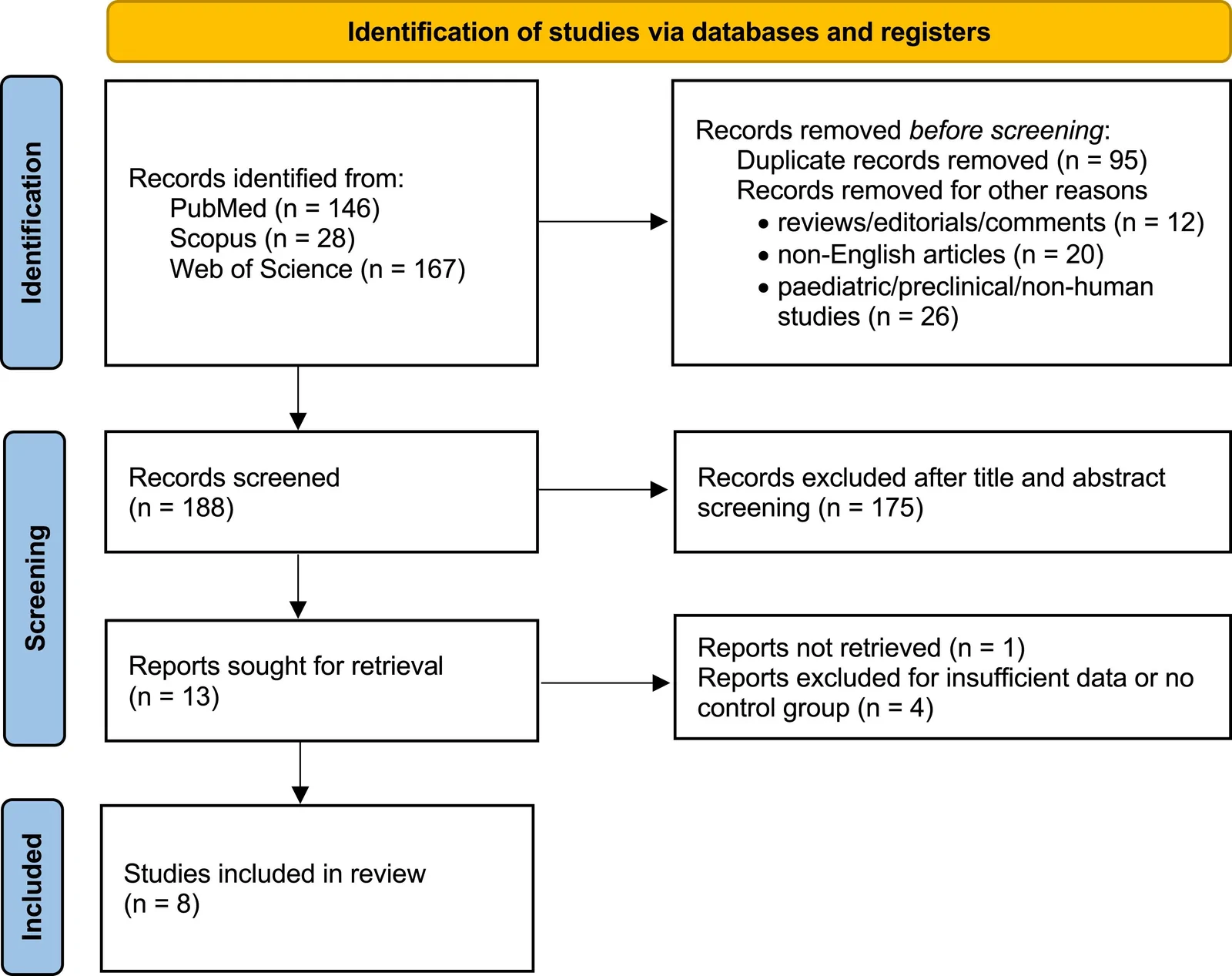
Flowchart of the study selection process for eligible studies.

### Data extraction

The reviewers independently extracted data from all included studies using full-text articles, tables, and supplementary materials. Extracted variables included study characteristics (authors, publication year, country, and study design), patient characteristics (sample size, sex, and age), type of autoimmune disease, diagnostic criteria for autoimmune disorders (including antibody positivity or clinically established disease), and the prevalence or risk estimates of autoimmune conditions in patients with KS compared with controls, when available. The main findings of the included studies are reported in the Results section.

### Statistical analysis

Random-effects models were prespecified given the expected clinical and methodological heterogeneity across study populations, autoimmune phenotypes, and outcome ascertainment strategies. Odds ratios (ORs) with corresponding 95% confidence intervals (CIs) were selected as the most appropriate summary measure given the predominantly dichotomous nature of autoimmune outcomes across included studies. Statistical heterogeneity was assessed using the I^2^ statistic, and 95% prediction intervals were calculated to estimate the expected range of effects in future studies. Heterogeneity was interpreted in the context of differences in autoimmune phenotypes, study design, and population characteristics rather than solely according to statistical thresholds. A sensitivity analysis was performed using a leave-one-out approach to evaluate the robustness of the pooled estimates. Publication bias was explored through funnel plot inspection and formally assessed using Egger's regression test when at least 10 studies were available, acknowledging the limited interpretability of small-study analyses in sparse meta-analytic datasets (20). Subgroup analyses were prespecified according to outcome definition (isolated autoantibody positivity vs clinically manifest autoimmune disease), given their potentially distinct biological and clinical implications. To minimize duplication bias, potentially overlapping cohorts were carefully evaluated and only the most comprehensive or methodologically informative dataset was retained for pooled analyses. Because several studies adopted reverse-ascertainment designs (ie, autoimmune disease cohorts screened for XXY aneuploidy rather than KS cohorts evaluated for autoimmune outcomes), datasets were evaluated for methodological comparability before quantitative synthesis. Studies were included in the meta-analysis only when sufficient comparative data were available to derive effect estimates reflecting autoimmune susceptibility associated with KS or XXY aneuploidy. Studies providing mechanistic or descriptive evidence without extractable comparative estimates were retained for qualitative synthesis only. All analyses were conducted using RStudio (version 2025.05.1+513).

## Results

### Literature search

A total of 341 records were identified through the electronic literature search. After duplicate removal and exclusion of reviews, non-English articles, and paediatric/preclinical studies, 188 records underwent title and abstract screening, of which 175 were excluded as not relevant to the review topic. Thirteen full-text reports were assessed for eligibility. One full-text article could not be retrieved, whereas 4 studies were excluded because of non-extractable KS-specific autoimmune outcome data or the absence of an appropriate control group (21). In total, eight studies were included in the review (10, 22-28) (Fig. 1). Most studies originated from Europe, followed by North America and multicentre international collaborations.

Overall, study quality assessed using the Newcastle–Ottawa Scale was moderate to high across the included studies, with NOS scores ranging from 7 to 8 points. Most studies showed adequate outcome ascertainment and appropriate definition of autoimmune conditions.

The main characteristics of the studies and their results are briefly presented in Tables 1 and 2.

**Table 1:** Characteristics of the studies considered for the review

First author	Ref. N.	Year	Country	Study design	Sample size	Age
Oktenli	(22)	2002	Turkey	Cross-sectional case–control study	67	22 ± 1 years
Seminog	(10)	2015	United Kingdom	National record-linkage cohort study	Not reported	Not reported
Harris	(23)	2016	Multicentre	Multicentre case–control study	2090	Not reported
Panimolle	(24)	2016	Italy	Cross-sectional case–control study	183	35 ± 11 years
Panimolle	(25)	2018	Italy	Cross-sectional case–control study	238	35 ± 11 years
Panimolle	(26)	2021	Italy	Cross-sectional case–control study	174	35 ± 11 years
Scofield	(27)	2022	Multicentre	Multicentre genetic association study	1580	Not reported
Palmer	(28)	2025	United States	Multicentre retrospective cohort study	51 245 146	Not reported

Abbreviations: N, number; Ref, references.

**Table 2:** Results and main findings of the studies considered for the review

First author	Karyotype	Autoimmune disease	Diagnostic criteria	Main findings	NOS
Harris (23)	47,XXY	Primary Sjögren syndrome, SLE, rheumatoid arthritis	AECG classification criteria	47,XXY significantly enriched among men with primary Sjögren syndrome and SLE.	8
Oktenli (22)	47,XXY	Systemic autoimmunity, thyroid autoimmunity	ANA, anti-dsDNA, ASMA, AMA, anti-ENA, anti-TPO, ICA, aCL positivity	Increased anti-ENA and anticardiolipin antibody positivity in KS patients	7
Palmer (28)	47,XXY	SLE, Sjögren disease	ICD-10 diagnosis codes	SLE and Sjögren disease significantly more frequent in KS patients	8
Panimolle (24)	47,XXY	Type 1 diabetes, Hashimoto thyroiditis, Addison disease, autoimmune gastritis	GAD, IA-2, ZnT8, insulin, TPO, 21-OH, APC positivity	Increased endocrine humoral autoimmunity, particularly diabetes-specific autoantibodies	7
Panimolle (25)	47,XXY and higher-grade aneuploidies	Type 1 diabetes, Hashimoto thyroiditis, Addison disease, autoimmune gastritis	GAD, IA-2, ZnT8, insulin, TPO, 21-OH, APC positivity	Higher frequency of endocrine organ-specific autoimmunity, increasing with X chromosome polysomy severity	7
Panimolle (26)	47,XXY and higher-grade aneuploidies	Systemic autoimmune diseases	ANA, ENA, ASMA, AMA, anti-dsDNA positivity	Increased non-organ-specific autoantibodies in KS patients compared with controls	7
Scofield (27)	47,XXY	Systemic sclerosis, polymyositis, dermatomyositis, inclusion body myositis	Subcommittee for Scleroderma Criteria of the American Rheumatism Association Diagnostic and Therapeutic Criteria Committee	Increased frequency of X chromosome aneuploidies in autoimmune rheumatic diseases	8
Seminog (10)	47,XXY	Multiple autoimmune diseases	Hospital admission/registry diagnosis codes	Increased risk of several autoimmune diseases, particularly female-predominant disorders	8

Abbreviations: aCL, anticardiolipin antibodies; AMA, anti-mitochondrial antibodies; ANA, antinuclear antibodies; anti-dsDNA, anti-double-stranded DNA antibodies; APC, anti-parietal cell antibodies; ASMA, anti-smooth muscle antibodies; ENA, extractable nuclear antigen antibodies; GAD, glutamic acid decarboxylase antibodies; IA-2, tyrosine phosphatase-related islet antigen 2 antibodies; ICA, islet cell antibodies; ICD-10, International Classification of Diseases, Tenth Revision; KS, Klinefelter syndrome; NOS, Newcastle–Ottawa Scale; RR, relative risk; SLE, systemic lupus erythematosus; TPO, thyroid peroxidase antibodies; ZnT8, zinc transporter 8 antibodies; 21-OH, 21-hydroxylase antibodies.

### Qualitative analysis

The included studies consistently supported an increased autoimmune burden in individuals with Klinefelter syndrome (KS), although the reported phenotypes ranged from isolated serological autoreactivity to clinically manifest systemic autoimmune diseases. Overall, the emerging autoimmune spectrum predominantly involved female-predominant and interferon-associated disorders, supporting the hypothesis that supernumerary X chromosome dosage may contribute to immune dysregulation through shared immune-regulatory pathways rather than nonspecific immune activation alone.

Evidence of endocrine and organ-specific humoral autoimmunity was reported across several studies. Oktenli et al demonstrated increased frequencies of anti-ENA antibodies (16.2% vs 3.3%) and anticardiolipin antibodies (29.7% vs 3.3%) in KS patients compared with healthy controls, suggesting enhanced humoral immune activation associated with hypogonadism and X chromosome polysomy (22). Similarly, Panimolle et al identified increased prevalences of diabetes-related autoantibodies, including GAD, IA-2, and ZnT8 antibodies, in adult 47,XXY individuals compared with controls, supporting a higher burden of endocrine humoral autoimmunity in KS (24). Increased frequencies of non-organ-specific autoantibodies, particularly ANA positivity, were also described in KS patients, further supporting the presence of broader immune dysregulation extending beyond endocrine autoimmunity (26).

Several studies additionally demonstrated enrichment of clinically overt systemic autoimmune diseases in KS, particularly disorders characterized by marked female predominance and type I interferon activation. Harris et al reported a significant excess of the 47,XXY karyotype among men with systemic lupus erythematosus and primary Sjögren syndrome (23). Similarly, Scofield et al identified increased frequencies of 47,XXY among patients with systemic sclerosis, polymyositis/dermatomyositis, and inclusion body myositis, reinforcing the association between XXY aneuploidy and interferon-associated autoimmune phenotypes (27).

Population-based evidence from English national record-linkage datasets further demonstrated increased risks of multiple autoimmune diseases in KS, including Addison disease, type 1 diabetes mellitus, autoimmune hypothyroidism, rheumatoid arthritis, Sjögren syndrome, and systemic lupus erythematosus (10). More recently, Palmer et al confirmed markedly increased prevalences of systemic lupus erythematosus and Sjögren disease in a large multicentre TriNetX cohort, further supporting the concept of a synergistic X chromosome dose effect in autoimmunity (28).

Collectively, the available evidence supports a biologically coherent autoimmune phenotype in KS characterized by enrichment of endocrine autoimmunity, interferon-associated systemic autoimmune diseases, and female-predominant immune-mediated disorders. These findings provide indirect support for the hypothesis that supernumerary X chromosome dosage may contribute directly to immune dysregulation and loss of self-tolerance in humans.

### Quantitative analysis

A total of eight datasets derived from six studies were included in the quantitative synthesis (22, 23, 25-28), comprising 2289 individuals with Klinefelter syndrome and 51 250 114 controls. All datasets included in the quantitative synthesis evaluated individuals with the classic 47,XXY karyotype. The quantitative synthesis demonstrated a substantially increased autoimmune susceptibility in individuals with KS compared with control populations (OR 23.25, 95% CI 9.96-54.31), with moderate-to-substantial between-study heterogeneity (I^2^ = 66.4%, τ^2^ = 0.7837, *P* = .0040) (Fig. 2). The 95% prediction interval remained compatible with increased autoimmune susceptibility (2.26-239.03), despite between-study heterogeneity. The magnitude of the pooled estimate should nevertheless be interpreted in the context of heterogeneous ascertainment strategies, inclusion of large registry-based datasets, and enrichment of autoimmune phenotypes in selected cohorts. Notably, between-study heterogeneity remained moderate-to-substantial despite important differences in autoimmune phenotypes, study design, and ascertainment strategies across the included studies. Sensitivity analyses confirmed the robustness of the pooled association across all leave-one-out iterations, with effect estimates remaining consistently significant throughout the analyses (OR range: 15.42-33.38; all *P* < .0001). Despite moderate between-study heterogeneity in the primary analysis, exclusion of individual datasets substantially attenuated heterogeneity in selected iterations without materially affecting the magnitude or direction of the association, supporting the overall stability and internal consistency of the findings (Fig. 3).

**Figure 2:**
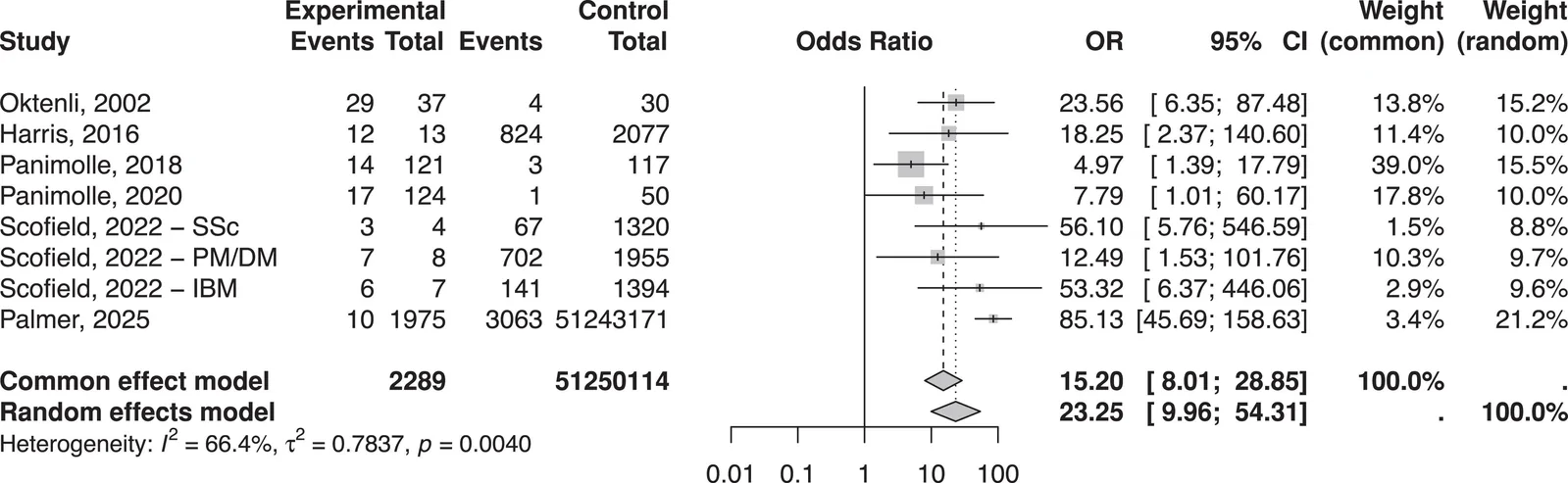
Forest plot showing the pooled association between Klinefelter syndrome and autoimmune diseases.

**Figure 3:**
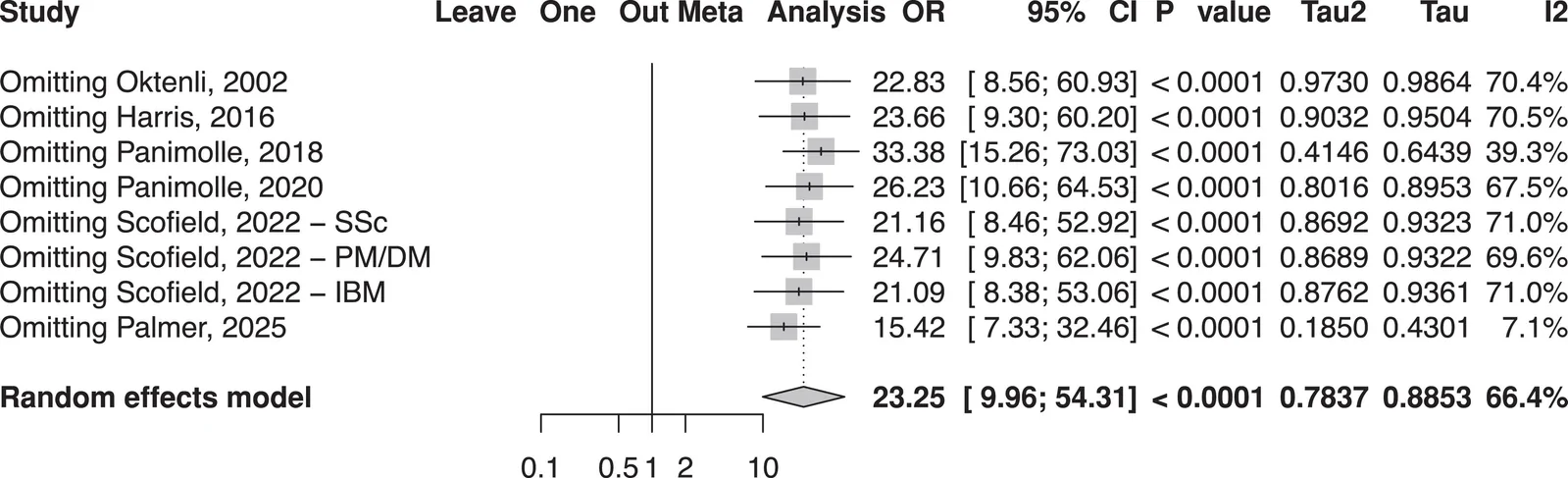
Leave-one-out sensitivity analysis evaluating the robustness of the association between Klinefelter syndrome and autoimmune susceptibility.

Because fewer than 10 datasets were included in the quantitative synthesis, formal assessment of publication bias using Egger's regression test was not performed, in accordance with current methodological recommendations. Nevertheless, visual inspection of the funnel plot did not suggest substantial asymmetry, although the possibility of small-study effects cannot be entirely excluded (Fig. 4).

**Figure 4:**
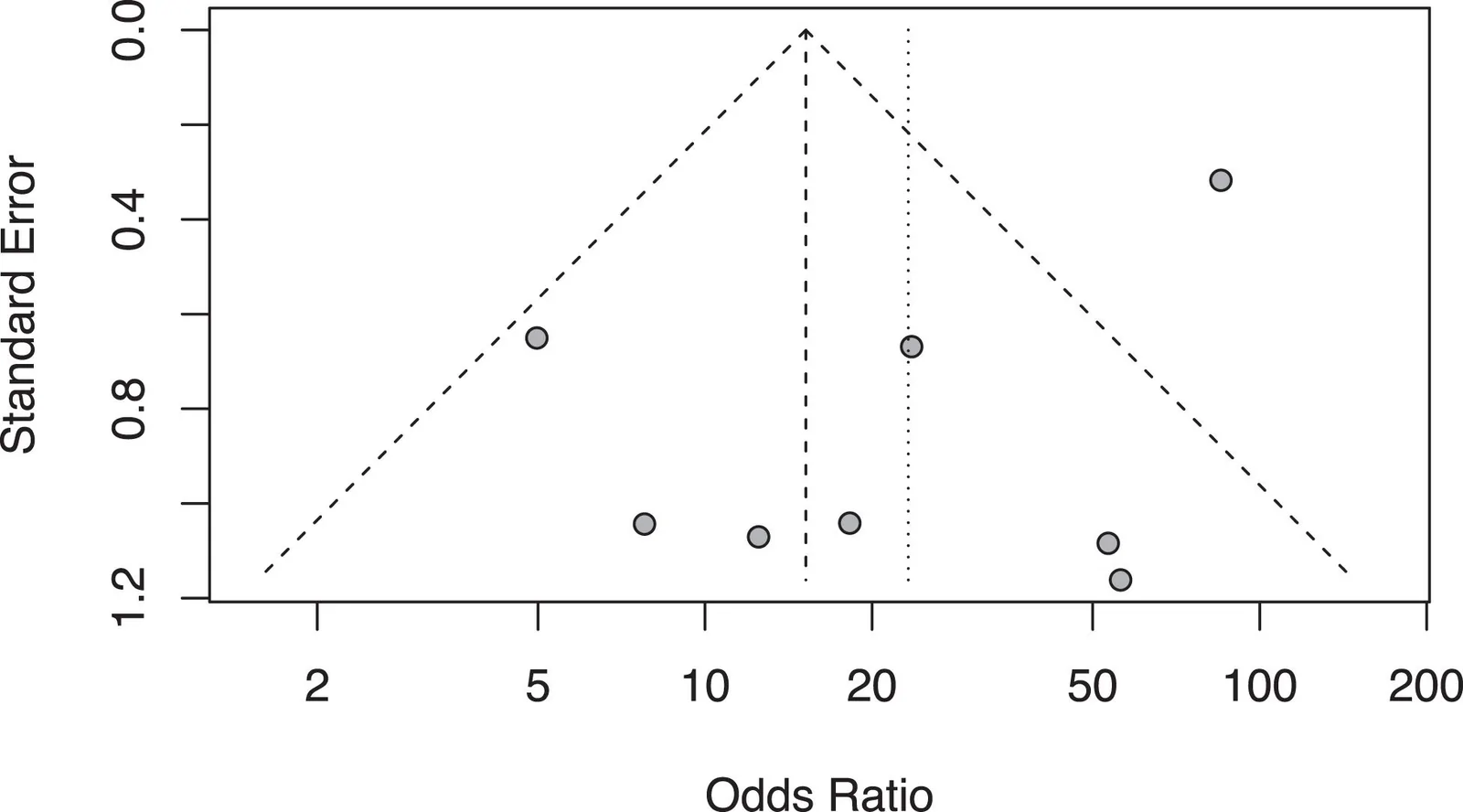
Funnel plot assessing potential publication bias in studies evaluating the association between Klinefelter syndrome and autoimmune diseases.

A subgroup analysis according to outcome definition was subsequently performed. Studies focused on isolated autoantibody positivity (22, 25, 26) demonstrated an increased autoimmune susceptibility in KS patients compared with controls (OR = 9.99, 95% CI 1.18-84.77), with low-to-moderate between-study heterogeneity (I^2^ = 29.9%, *P* = .2403). Conversely, studies evaluating clinically manifest autoimmune diseases (23, 27, 28) showed a stronger and more consistent association (OR = 48.46, 95% CI 17.68-132.87), with low between-study heterogeneity (I^2^ = 13.6%, *P* = .3274) (Fig. 5). This pattern suggests that XXY aneuploidy may be more strongly associated with progression toward clinically overt immune-mediated disease than with isolated serological autoreactivity alone.

**Figure 5:**
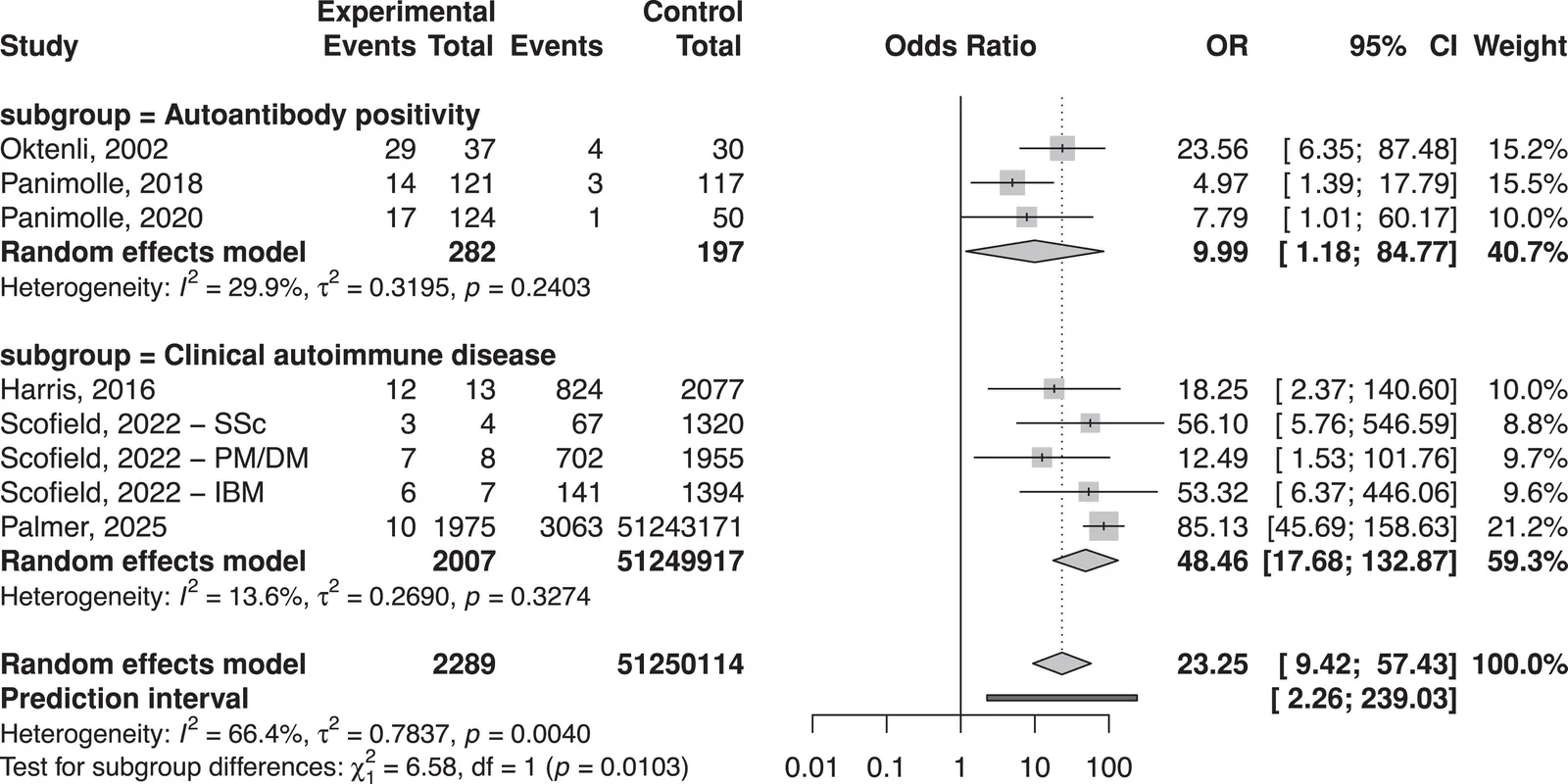
Subgroup analysis according to outcome definition in studies evaluating the association between Klinefelter syndrome and autoimmune susceptibility.

## Discussion

This systematic review and meta-analysis show that the classic 47,XXY karyotype is associated with a substantially increased autoimmune susceptibility and supports the concept that sex chromosome dosage contributes to immune dysregulation in humans. The association was particularly evident for clinically overt autoimmune disorders, whereas the relationship with isolated autoantibody positivity appeared less consistent. Accordingly, these findings should be interpreted as applying primarily to individuals with the classic 47,XXY karyotype. Collectively, these findings support the concept that KS represents not only a reproductive and endocrine disorder, but also a systemic condition characterized by substantial immune dysregulation.

The biological plausibility underlying the association between KS and autoimmunity is supported by converging evidence implicating both sex chromosome complement and endocrine dysfunction in immune regulation. Increasing data suggest that the marked female predominance observed across several autoimmune diseases cannot be explained solely by hormonal influences but also reflects intrinsic effects of sex chromosome dosage on immune and epigenetic regulation (14, 29, 30).

Among X-linked immune regulators, TLR7 represents a particularly compelling candidate mechanism linking supernumerary X chromosome dosage to interferon-driven autoimmunity. TLR7 is a key innate immune sensor involved in nucleic acid recognition and type I interferon activation, pathways strongly implicated in female-biased autoimmune diseases such as systemic lupus erythematosus and Sjögren syndrome (31-33). Experimental evidence indicates that TLR7 may escape X-chromosome inactivation in immune cells, potentially resulting in enhanced transcriptional activity in individuals carrying additional X chromosomes (31). This framework is further supported by murine lupus models, in which TLR7 overexpression directly amplifies autoimmune responses and accelerates systemic autoimmunity (34, 35).

Notably, the autoimmune spectrum emerging from the present review closely mirrors interferon-associated autoimmune phenotypes characterized by marked female predominance, including systemic lupus erythematosus, Sjögren syndrome, systemic sclerosis, and inflammatory myopathies. These observations support the hypothesis that increased X chromosome dosage may preferentially amplify specific immune-regulatory pathways rather than simply increasing autoimmune burden in a nonspecific manner. Additional X-linked immune regulators may further contribute to this process. FOXP3 plays a central role in regulatory T-cell differentiation and peripheral immune tolerance, whereas CD40L overexpression may enhance autoreactive B-cell activation and autoantibody production (36, 37). Overall, these findings support a broader biological framework in which sex chromosome complement directly contributes to immune homeostasis and loss of self-tolerance. Recent single-cell transcriptomic analyses further support this biological framework. Yazar et al identified widespread sex-biased immune gene expression and sex-specific regulatory variants across multiple immune cell populations, including pathways related to TNF-α signaling and genes previously implicated in autoimmune diseases (38). In this context, KS may represent a useful clinical framework for investigating the relationship between sex chromosome dosage and immune dysregulation. A proposed integrative framework linking supernumerary X chromosome dosage, interferon-associated immune activation, endocrine dysfunction, and female-predominant autoimmunity in KS is summarized in Fig. 6.

**Figure 6:**
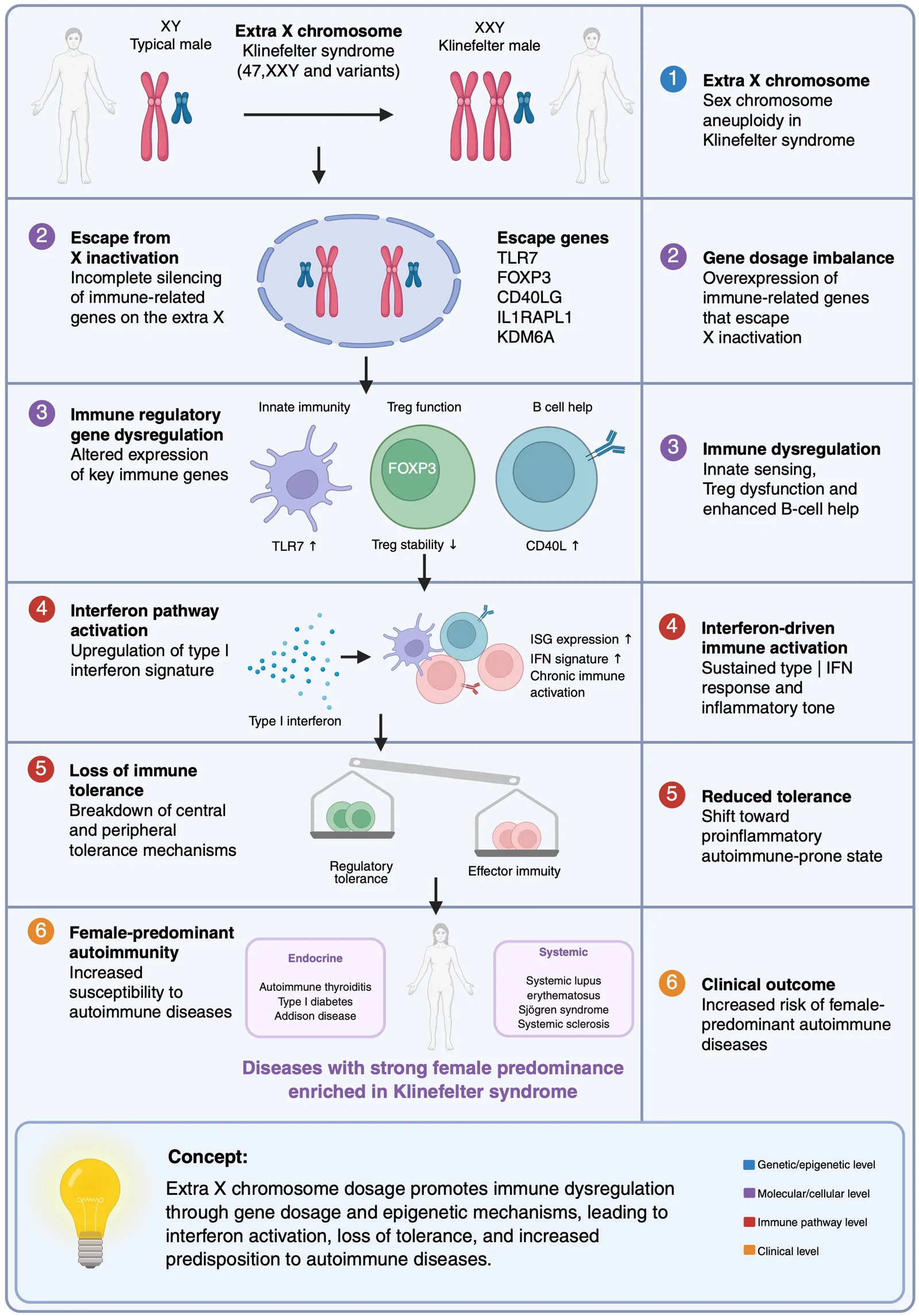
Proposed biological framework linking extra X chromosome dosage to immune dysregulation and female-predominant autoimmune susceptibility in Klinefelter syndrome.

Most of the mechanistic evidence discussed below derives from studies comparing 46,XX and 46,XY individuals rather than specifically investigating individuals with the classic 47,XXY karyotype. Accordingly, these observations should be interpreted as indirect biological support for the mechanisms potentially underlying the increased autoimmune susceptibility observed in Klinefelter syndrome. Consistent with this framework, XXY aneuploidy appears preferentially enriched in interferon-associated autoimmune diseases. Similar observations have also been reported in other sex chromosome abnormalities, particularly Turner syndrome, which has likewise been associated with increased susceptibility to autoimmune disorders despite profoundly different hormonal profiles (17, 18, 39). Together, these findings reinforce the concept that sex chromosome complement exerts biologically relevant effects on immune regulation independent of gonadal phenotype alone. Moreover, previous observations suggesting a higher prevalence of autoimmune manifestations in individuals with more complex sex chromosome polysomies raise the possibility of a progressive gene-dose effect linking X chromosome burden to immune dysregulation (10). This hypothesis is also supported by the observations of Panimolle et al, who reported numerically higher frequencies of endocrine autoantibody positivity in individuals with higher-grade X chromosome aneuploidies compared with those carrying the classic 47,XXY karyotype, although these findings require confirmation in larger cohorts (25).

The relationship between hypogonadism, sex chromosome dosage, and immune dysregulation in KS is likely to be biologically interconnected rather than mechanistically distinct. Although androgen deficiency has traditionally been considered a potential contributor to autoimmune susceptibility, the female-predominant autoimmune phenotype observed in XXY individuals suggests that hormonal imbalance alone is unlikely to fully explain the immune profile associated with KS (29, 40, 41). Experimental and translational evidence increasingly indicates that sex chromosome complement and hormonal milieu exert both joint and independent effects on immune and tissue-specific gene regulation (13). In this context, endocrine dysfunction and supernumerary X chromosome dosage may converge on shared immune-regulatory pathways contributing to systemic autoimmunity in KS rather than representing competing explanatory mechanisms.

Importantly, the stronger association observed for clinically manifest autoimmune diseases compared with isolated autoantibody positivity may suggest that XXY aneuploidy influences not only early immune dysregulation, but also the transition toward clinically overt autoimmune disease. Accordingly, supernumerary X chromosome dosage may contribute not only to autoreactive immune signatures, but also to the emergence of clinically relevant autoimmune phenotypes. Clinical observations partially support this integrated framework. Bizzarro et al reported improvements in both immunological parameters and clinical manifestations following testosterone replacement therapy in patients with KS-associated autoimmune diseases, including reductions in ANA and rheumatoid factor titers together with normalization of altered T-cell subsets (42). Similarly, Panimolle et al observed numerically lower frequencies of endocrine autoantibody positivity in testosterone-treated KS patients compared with untreated individuals, although statistical significance was not reached (25).

The present study has several strengths. To our knowledge, this is the first quantitative synthesis specifically evaluating autoimmune diseases in KS. The study integrates evidence across both endocrine and systemic autoimmune disorders, incorporates subgroup and sensitivity analyses, and adopts a conservative strategy to minimize cohort overlap. Moreover, the distinction between clinically overt autoimmune disease and isolated serological autoimmunity provides additional insight into the potential biological relevance of the observed associations.

Several limitations should nevertheless be acknowledged. First, the number of available studies remained limited for several autoimmune outcomes, and many included studies were observational and retrospective, precluding causal inference. Second, clinical and methodological heterogeneity remained present across included studies, likely reflecting differences in study design, ascertainment strategies, population characteristics, and definitions of autoimmune outcomes. Several included studies were additionally susceptible to ascertainment and surveillance biases inherent to registry-based and reverse-ascertainment designs. Third, some pooled estimates were heavily influenced by large registry-based cohorts, whereas smaller studies frequently lacked sufficient statistical power. Finally, the available evidence did not allow precise dissection of the relative contribution of endocrine dysfunction, sex chromosome dosage, and environmental factors to autoimmune burden in KS. Accordingly, the proposed mechanistic framework should be interpreted as biologically plausible rather than definitively causal.

In conclusion, KS is associated with an increased prevalence of autoimmune diseases, particularly clinically manifest autoimmune disorders. These findings support a contribution of sex chromosome complement and endocrine dysfunction to immune regulation and reinforce the importance of clinical awareness of autoimmune comorbidities in KS.

The protocol of this systematic review was registered in PROSPERO (CRD420261399097).

## Data Availability

Original data generated and analyzed during this study are included in this published article.
